# Efficacy of locally-delivered statins adjunct to non-surgical periodontal therapy for chronic periodontitis: a Bayesian network analysis

**DOI:** 10.1186/s12903-019-0789-2

**Published:** 2019-06-13

**Authors:** Ruoyan Cao, Qiulan Li, Yu Chen, Mianfeng Yao, Qiqi Wu, Hongbo Zhou

**Affiliations:** 1 Department of Prosthodontics, Xiangya Stomatological Hospital & School of Stomatology, Central South University, 72 Xiangya Road, Changsha, 410000 China; 2 Department of Stomatology, The Second Xiangya Hospital, Central South University, 139 Middle Renmin Road, Changsha, 410011 China; 3 Department of Oral Medicine, Xiangya Hospital, Central South University, 87 Xiangya Road, Changsha, 410083 China; 4 Department of Operative Dentistry and Endodontics, Stomatological Hospital & School of Stomatology, Central South University, 72 Xiangya Road, Changsha, 410000 China

**Keywords:** Statins, Chronic periodontitis, Periodontal therapy, Network meta-analysis

## Abstract

**Background:**

Studies indicate locally-delivered statins offer additional benefits to scaling and root planning (SRP), however, it is still hard to say which type of statins is better. This network meta-analysis aimed to assess the effect of locally-delivered statins and rank the most efficacious statin for treating chronic periodontitis (CP) in combination with SRP.

**Methods:**

We screened four literature databases (Pubmed, Embase, Cochrane Library, and Web of Science) for randomized controlled clinical trials (RCTs) published up to June 2018 that compared different statins in the treatment of chronic periodontitis. The outcomes analyzed were changes in intrabony defect depth (IBD), pocket depth (PD), and clinical attachment level (CAL). We carried out Bayesian network meta-analysis of CP without systemic diseases. Traditional and Bayesian network meta-analyses were conducted using random-effects models.

**Results:**

Greater filling of IBD, reduction in PD, and gain in CAL were observed for SRP treated in combination with statins when compared to SRP alone for treating CP without systemic diseases. Specifically, SRP+ Atorvastatin (ATV) (mean difference [MD]: 1.5 mm, 1.4 mm, 1.8 mm, respectively), SRP + Rosuvastatin (RSV) (MD: 1.8 mm, 2.0 mm, 2.1 mm, respectively), and SRP + Simvastatin (SMV) (MD: 1.1 mm, 2.2 mm, 2.1 mm, respectively) were identified. However, no difference was found among the statins tested. In CP patients with type 2 diabetic (T2DM) or in smokers, additional benefits were observed from locally delivered statins.

**Conclusion:**

Local statin use adjunctive to SRP confers additional benefits in treating CP by SRP, even in T2DM and smokers. RSV may be the best one to fill in IBD. However, considering the limitations of this study, clinicians must use cautious when applying the results and further studies are required to explore the efficacy of statins in CP with or without the risk factors (T2DM comorbidity or smoking history).

**Electronic supplementary material:**

The online version of this article (10.1186/s12903-019-0789-2) contains supplementary material, which is available to authorized users.

## Background

Chronic periodontitis (CP) is a multifactorial inflammatory disease caused by pathogenic microorganisms and disordered host immune inflammation that leads to bone resorption, bony defects, and ultimately tooth loss [1]. Nonsurgical periodontal treatment reduces pocket depth (PD) and increases clinical attachment level (CAL) to some extent [2, 3], but fails to fill the bony defect [4]. Thus, various adjuvant therapies have been applied in nonsurgical treatment to reduce tissue destruction and to enhance periodontal reparative processes including statins.

Statins are inhibitors of 3-hydroxy-3-glutaryl-coenzyme A reductase and are primarily used to prevent hyperlipidemia and coronary artery disease [5, 6]. However, with in-depth study of statins, additional benefits have been found in the treatment of periodontal diseases. This phenomenon may be due to the unique properties of statins that limit the pathogenesis of periodontitis, such as anti-inflammatory [7, 8], anti-microbial [9], bone formation promoting, bone loss inhibiting [10, 11] and antioxidant properties [12]. Different statins exhibit different such properties, which could lead to different treatment outcomes. For example, rosuvastatin (RSV) is thought to possess stronger anti-inflammatory potential than atorvastatin (ATV) [13], while ATV is stronger than simvastatin (SMV) in terms of anti-inflammatory and antioxidant potential [14, 15]. SMV is considered to be the optimal statin for controlling periodontal pathogens, such as *Porphyromonas gingivalis* (*Pg*) and *Aggregatibacter actinomycetemcomitans* (*Aa*) [16]. However, clinical trials investigating the effects of different statins on adjuvant treatment of CP are limited. To our knowledge, there are six meta-analyses comparing statins adjunctive to scaling and root planing (SRP) with SRP alone, however, they fail to measure the relative effects of various statins on CP without other systemic diseases [4, 17–21]. Therefore, a network meta-analysis which compares and ranks different statins should be beneficial to clinical practice.

This network meta-analysis aimed to study whether local statins applied adjunctively to nonsurgical periodontal treatment contribute to better clinical and histological periodontal outcomes based on randomized controlled clinical trials (RCTs) when compared to periodontal treatment alone in patients with CP. This study further ranked statins based on their adjunct efficacy with SRP.

## Methods

### Protocol registration

This meta-analysis was prospectively registered at the National Institute for Health Research PROSPERO, International Prospective Register of Systematic Reviews (http://www.crd.york.ac.uk/PROSPERO, registration no.: CRD42018100753).

### Inclusion criteria

Only RCTs followed up for at least 6 months were included in this network meta-analysis. PICO criteria was defined as [22]:

- (P) Participants: Patients with chronic periodontitis without periodontal therapy and use of antibiotics in the past 6 months.
- (I) Interventions: The following locally delivered statins employed adjunctively to periodontal treatment were considered: SRP + ATV, SRP + SMV, and SRP + RSV
- (C) Comparison: SRP alone
- (O) Outcome measures: primary outcome: changes in IBD; secondary outcomes: changes in PD and CAL

### Exclusion criteria

Studies that had any of the following characteristics were excluded: (a) split-mouth RCT design; (b) inclusion patients with statin allergy; (c) application of systemic statin therapy; (d) inclusion of immunocompromised individuals; (e) inclusion of former smokers; (f) systemic diseases except for type 2 diabetes.

### Search methods for study identification

To identify RCTs for this network meta-analysis, we searched the Pubmed, Embase, Cochrane Library, and Web of Science databases for relevant publications published up to June 2018. The following MeSH terms/free terms and their combinations searched are described in Additional file 1. The resulting reference lists of relevant articles and relevant systematic reviews [4, 17–20] was manually screened to find other potentially eligible studies.

### Data collection, extraction and management

Two researchers (R.Y.Cao & Q.L.Li) independently screened the databases search for relevant titles and abstracts. Then data was extracted and recorded relevant information from eligible studies with pre-designed data-extraction forms using the following criteria: surname of the first author, publication year, country, characteristics of participants (age, gender, smoking status, systemic diseases), sample size, type of interventions, number of application sites/patients, application mode/site, application period, periodontal probe, outcome (IBD, PD, CAL, baseline and mean change in parameters from baseline to follow-up visits). Disagreements on study inclusion or data extraction were resolved through discussion among the researchers. When necessary, a third investigator (M.F.Yao) helped to reach a consensus with all reviewers.

### Risk of bias assessment

The risk of bias of the included studies was performed independently by two researchers (R.Y.Cao & Q.L.Li) using the Cochrane Collaboration tool [23]. Any disagreements were solved by the third investigator (M.F.Yao).

### Statistical analysis

The treatment outcomes were measured as the absolute difference (AD) in IBD, PD, and CAL in at least 6 months after periodontal treatment. When standard deviations (SD) for the outcomes parameters were not available, they were calculated by assuming the correlation coefficient to be 0.5 as previously described [24]. Based on patient characteristics, the studies were divided into three subgroups (systemic healthy, T2DM, and smokers). Network meta-analysis was only applied to the systemic healthy subgroup as there were two studies in other subgroups. The same follow-up duration was used in this meta-analysis in the subgroups.

First, we developed a random-effects pairwise meta-analysis in Stata 14.2 (Stata Corporation, College Station, TX, USA). Weighted mean difference (WMD) and 95% confidence intervals (CIs) were used to compare continuous variables. Second, Bayesian network meta-analyses were performed by using a random-effect model to pool the effect sizes of both direct and indirect comparisons. Non-informative uniform and normal prior distributions were used throughout the network meta-analysis. Markov chain Monte Carlo methods with four chains of 300,000 iterations after a burn-in phase of 100,000 iterations was performed to achieve credible mean difference (MD) and 95% credible intervals (CrIs). We used CrIs beyond the null value to assess significance and ranked different treatments.

Inconsistency was assessed by comparing direct evidence with indirect evidence from the entire network at each node (node-splitting analysis) with *p* < 0.05 [25]. Moreover, we examined the pooled effects from traditional pairwise meta-analysis and network meta-analysis to further verify the consistency of the network. The goodness of fit of the model was tested by calculating the posterior mean residual deviance (Dbar). When the Dbar was similar to the number of data points in the study, the model was considered to fit the data well [26, 27]. Heterogeneity was assessed with I^2^ calculation. Sensitivity analysis was performed to verify the robustness of our analyses by excluding studies with a high risk of bias then the effect was recalculated. R 3.2.2 (R Foundation for Statistical Computing, Vienna, Austria) GeMTC 0.8 package was used to analyse all data.

## Results

**Fig. 1:**
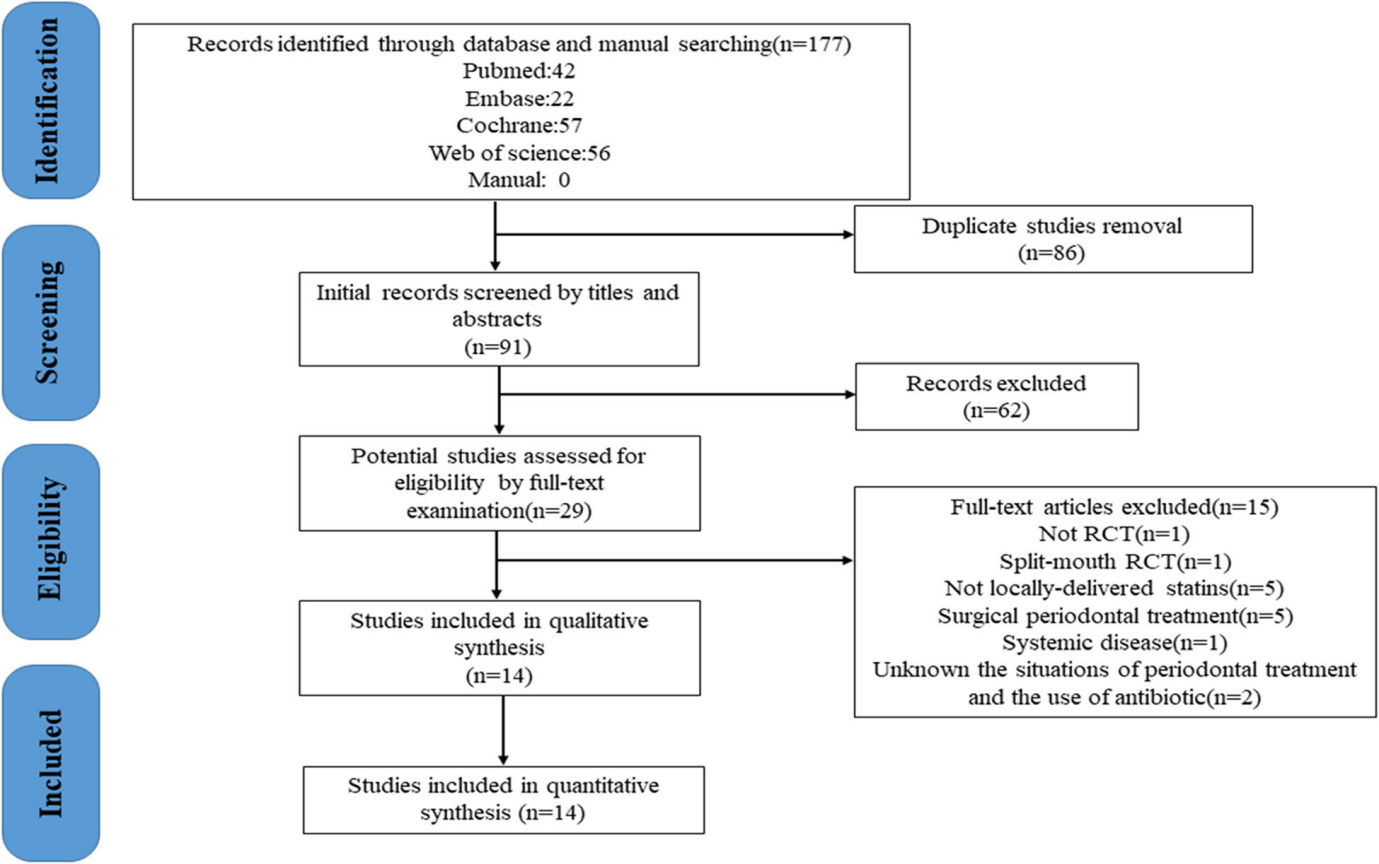
Flowchart of articles search and screening process

Study selection and the exclusion criteria are summarized in Fig. 1. A total of 126 citations were obtained for title and abstract review. Finally, 14 studies were selected for inclusion that met all the inclusion criteria and were analyzed in a pair-wise meta-analysis. Ten of studies were included in network meta-analysis.

### Studies characteristics

**Fig. 2:**
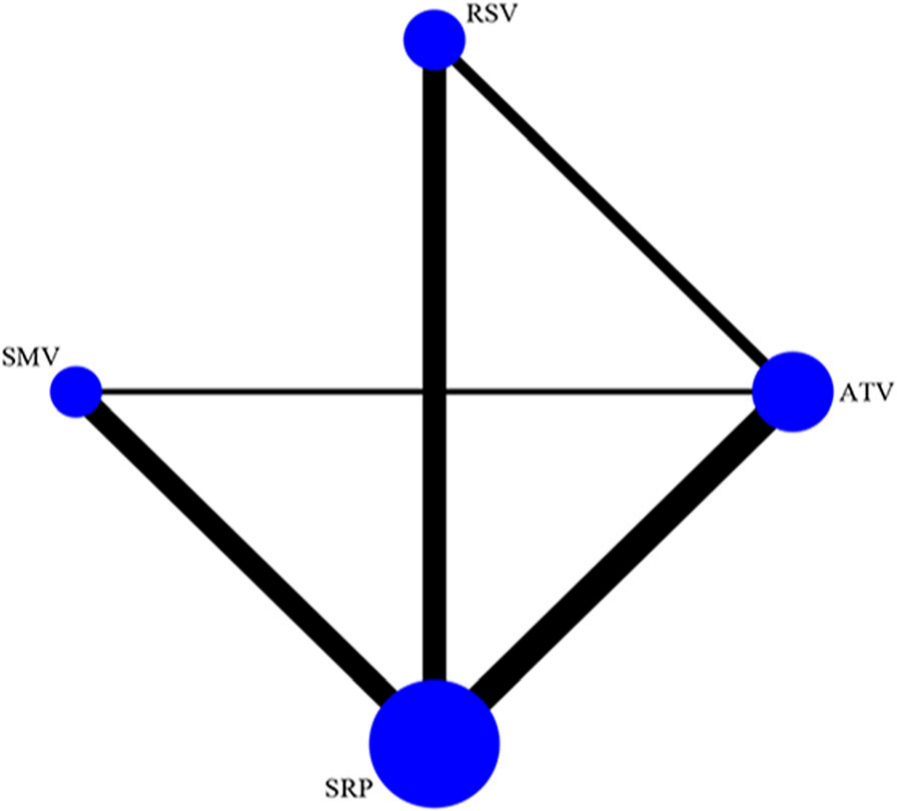
Network of the interventional comparisons for the Bayesian network analysis. The size of the nodes is proportional to the number of subjects (sample size) randomized to receive the therapy. The width of the lines is proportional to the number of trials comparing each pair of treatments. SRP, scaling and root planing; SMV, simvastatin; ATV, atorvastatin; RSV, rosuvastatin

Table 1 and Additional file 2 present the main characteristics of the included studies, all of which were parallel RCTs with a follow-up of 6–9 months. Ten trials included patients without systemic diseases and excluded smokers [28–31, 34, 36, 37, 39–41], two trials concerned patients with T2DM [32, 35] or smokers [33, 38]. All included studies reported clear inclusion criteria and patients with periodontitis had similar intrabony defects, PD, and CAL in each subgroup. All studies employed a 1.2% statin gel with a dose of 0.1 ml or 10 μl. In two studies [36, 37], the statin gel was applied after SRP and re-applied again 6 months after while others applied statin gel only once after SRP. There were no adverse events observed in all trials. Figure 2 shows the weighted network.

**Table 1:** Characteristics of studies included in the network meta-analysis

Study (year)	Country	Study design	Inclusion criteria	Age	Female/male	Smoking status Systemic diseases	Intervention (T vs. C)	No. of application sites (S)/patients (P)	Application mode/site	Application period (days)
Pradeep (2010) [28]	India	RCT	Intrabony defects, moderate (PD: 5–6 mm or CAL:4–6 mm; *n* = 24) to deep pockets (PD ≥ 7 mm or CAL:6–9 mm; *n* = 36) and vertical BL ≥ 3 mm	30.5 ± 4.1	31/33	No	SRP + SMV gel (1.2 mg/0.1 ml, 0.1 ml) (*n* = 30) vs. SRP + placebo gel (*n* = 30)	1S/P	Subgingival	1
Pradeep (2012) [29]	India	RCT	Furcation defects (buccal Class II, mandibular first and second molars), PD ≥ 5 mm and horizontal PD ≥ 3 mm	30–50	34/38	No	SRP + SMV gel (1.2 mg/0.1 ml, 0.1 ml) (*n* = 33) vs. SRP + placebo gel (*n* = 33)	1S/P	Furcation defect	1
Rath (2012) [30]	India	RCT	Intrabony defects, PD > 5 mm and vertical BL ≥ 3 mm	25–45	27/33	No	SRP + SMV gel (1.2 mg/0.1 ml, 0.1 ml) (*n* = 30) vs. SRP + placebo gel (*n* = 30)	1S/P	Subgingival	1
Pradeep (2013a) [31]	India	RCT	Intrabony defects, PD ≥ 5 mm or CAL ≥ 4 mm and vertical BL ≥3 mm	30–50	32/35	No	SRP + ATV gel (1.2 mg/0.1 ml, 10ul) (*n* = 30) vs. SRP + placebo gel (*n* = 30)	1S/P	Subgingival	1
Pradeep (2013b) [32]	India	RCT	Intrabony defects, PD ≥ 5 mm or CAL ≥ 4 mm and vertical BL ≥ 3 mm	30–50	18/20	Type 2 diabetes	SRP + SMV gel (1.2 mg/0.1 ml, 10ul) (*n* = 29) vs. SRP + placebo gel (*n* = 29)	29S/17P vs. 29S/18P	Subgingival	1
Rao (2013) [33]	India	RCT	Intrabony defects, PD ≥ 5 mm or CAL ≥ 4 mm and vertical BL ≥ 3 mm	30–50	Not report	Smokers (> 10 cigarettes/day for at least 5 years)	SRP + SMV gel (1.2 mg/0.1 ml, 10ul) (*n* = 33) vs. SRP + placebo gel (*n* = 34)	33S/17P vs. 34S/18P	Subgingival	1
Pradeep (2015) [34]	India	RCT	Intrabony defects, moderate (PD: 5–6 mm or CAL:4–6 mm; *n* = 24) to deep pockets (PD ≥ 7 mm or CAL:6–9 mm; *n* = 36) and vertical BL ≥ 3 mm	25–50	37/33	No	SRP + RSV gel (1.2 mg/0.1 ml, 0.1 ml) (*n* = 32) vs. SRP + placebo gel (*n* = 33)	1S/P	Subgingival	1
Kumari (2016) [35]	India	RCT	Intrabony defects, PD ≥ 5 mm or CAL ≥ 4 mm and vertical BL ≥ 3 mm	40–50	37/38	Type 2 diabetes	SRP + ATV gel (1.2 mg/0.1 ml, 10ul) (*n* = 30) vs. SRP + placebo gel (*n* = 30)	1S/P	Subgingival	1
Pradeep (2016) [36]	India	RCT	Intrabony defects, PD ≥ 5 mm, CAL ≥ 4 mm and vertical BL ≥ 3 mm	25–45	45/45	No	I: SRP + RSV gel (1.2 mg/0.1 ml, 0.1 ml) (*n* = 27) II: SRP + ATV gel (1.2 mg/0.1 ml, 0.1 ml) (*n* = 27) III: SRP + placebo gel (*n* = 27)	1S/P	Subgingival	1 and 180 (after 6 m re-deliver)
Garg (2017) [37]	India	RCT	Mandibular Class II furcation defects, PD ≥5 mm and horizontal PD ≥ 3 mm	30–50	Not report	No	I: SRP + RSV gel (1.2 mg/0.1 ml,0.1 ml) (*n* = 30) II: I: SRP + ATV gel (1.2 mg/0.1 ml,0.1 ml) (*n* = 30) III: SRP + placebo gel (*n* = 30)	1S/P	Subgingival	1 and 180 (after 6 m re-deliver)
Kumari (2017) [38]	India	RCT	Intrabony defects, PD ≥ 5 mm or CAL ≥ 4 mm and vertical BL ≥ 3 mm	30–50	Not report	Smokers (> 10 cigarettes/day for at least 5 years)	SRP + ATV gel (1.2 mg/0.1 ml, 10ul) (*n* = 33) vs. SRP + placebo gel (*n* = 33)	1S/P	Subgingival	1
Pradeep (2017) [39]	India	RCT	Intrabony defects, PD ≥ 5 mm or CAL ≥ 4 to 6 mm and vertical BL ≥ 3 mm	30–50	Not report	No	SRP + ATV gel (1.2 mg/0.1 ml, 10ul) (*n* = 30) vs. SRP + placebo gel (*n* = 30)	1S/P	Subgingival	1
Martande (2017) [40]	India	RCT	Intrabony defects, PD ≥ 5 mm or CAL ≥ 4 mm and vertical BL ≥ 3 mm	30–50	46/50	No	SRP + ATV gel (1.2 mg/0.1 ml, 10ul) (*n* = 30) vs. SRP + SMV gel (1.2 mg/0.1 ml, 10ul) (*n* = 30) vs. SRP + placebo gel (*n* = 28)	1S/P	Subgingival	1
Dileep P (2018) [41]	India	RCT	Intrabony defects, PD ≥ 5 mm and CAL > 3 mm	24–41	29/31	No	SRP + RSV gel (1.2 mg/0.1 ml, 0.1 ml) (*n* = 30) vs. SRP + placebo gel (*n* = 30)	1S/P	Subgingival	1

*SRP* Scaling and root planing, *SMV* Simvastatin, *ATV* Atorvastatin, *RSV* Rosuvastatin, *T* Treatment, *C* Control, *PD* Probing depth, *CAL* Clinical attachment loss, *BL* Bone loss

### Risk of bias in included studies

Additional file 3 details the quality of each of the 14 RCTs. All the trials described the methods of sequence generation, two trials used coin toss [28, 30], and the rest used a computer-generated random table. Seven trials employed allocation concealment [31–35, 37, 38]. All studies reported whether participants or study personnel were blinded, and three studies [30, 40, 42] did not report whether these groups were blinded to outcome assessment. After considering such little incomplete outcome data, reporting bias and other bias domains, all studies had a low risk of bias.

### Synthesis of results

#### Effects of statins in subjects without systemic diseases

Ten trials were included for CP without systemic diseases, and the results of standard pairwise meta-analysis and network meta-analysis are presented (Additional file 4, Fig. 3). Changes in periodontal parameters (IBD, PD, and CAL) were significant higher in SRP + SMV/ATV/RSV group than in SRP group alone in both pairwise and network meta-analysis. No significant difference was found in the changes in IBD, PD, and CAL in network meta-analysis. RSV was ranked as the best statin in terms of IBD outcomes while SMV ranked the best for PD and CAL outcomes (Fig. 4). Network meta-analysis showed considerable heterogeneity with global I^2^ > 90 (Table 2).

**Fig. 3:**
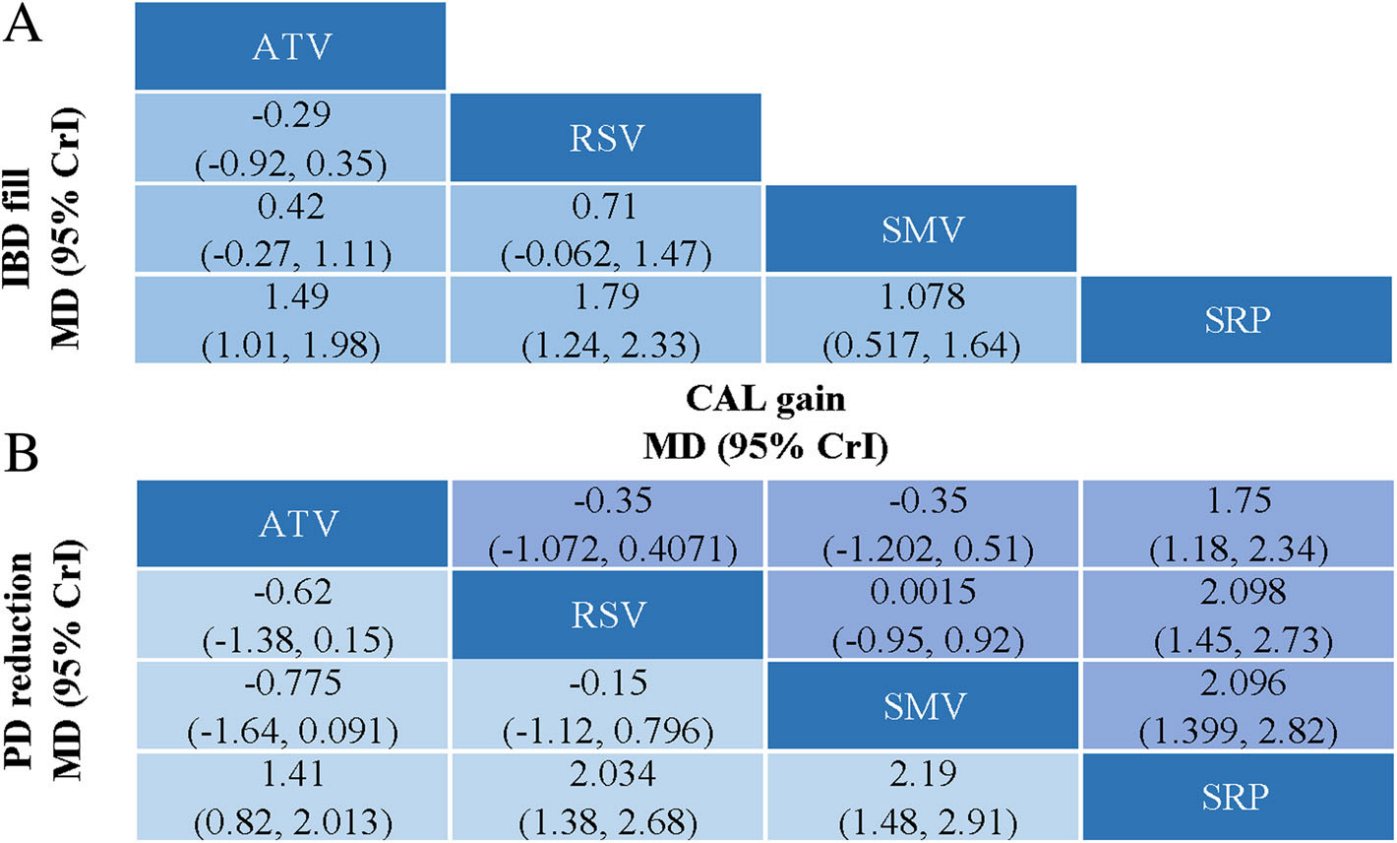
Multiple-treatment comparisons for ΔPD, ΔCAL, IBD fill in CP without systemic diseases. PD, probing depth; CAL, clinical attachment loss; IBD, intrabony defect; SRP, scaling and root planing; SMV, simvastatin; ATV, atorvastatin; RSV, rosuvastatin

**Fig. 4:**
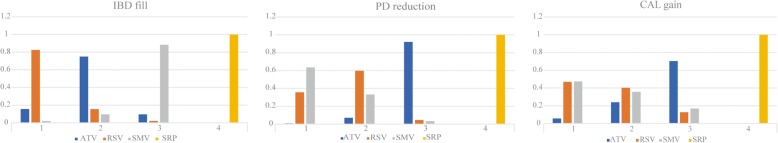
The rank of different treatments. PD, probing depth; CAL, clinical attachment loss; IBD, intrabony defect; SRP, scaling and root planing; SMV, simvastatin; ATV, atorvastatin; RSV, rosuvastatin

**Table 2:** Analysis of heterogeneity

t1	t2	i2.pair	i2.cons	incons.p
PD				
Per-comparison I-squared				
ATV	SRP	86.12230	86.70305	NA
RSV	SRP	97.77175	97.30109	NA
SMV	SRP	73.65906	76.46786	NA
ATV	RSV	0.00000	25.43579	0.45083148
ATV	SMV	NA	88.02565	0.08914125
Global I-squared		93.72891	92.04248	
CAL				
Per-comparison I-squared				
ATV	SRP	90.50355	91.37052	NA
RSV	SRP	99.36135	99.10428	NA
SMV	SRP	12.06329	13.87502	NA
ATV	RSV	0.00000	53.34930	0.3627355
ATV	SMV	NA	68.13125	0.2851176
Global I-squared		97.86568	96.677	
IBD				
Per-comparison I-squared				
ATV	SRP	95.00764	94.65418	NA
RSV	SRP	97.78225	97.79998	NA
SMV	SRP	83.90910	82.98919	NA
ATV	RSV	0.00000	0.00	0.8532051
ATV	SMV	NA	0.00000	0.6941173
Global I-squared		95.30703	94.33677	

*PD* Probing depth, *CAL* Clinical attachment loss, *IBD* Intrabony defect, *SMV* Simvastatin, *ATV* Atorvastatin, *RSV* Rosuvastatin, *t1* Treatment 1, *t2* Treatment 2, *i2.pair* i-square of pair-wise meta-analysis, *i2.cons* i-square of network meta-analysis, *incons.p* inconsistency *p*-values for pairwise and network meta-analysis, *NA* Not applicable

#### Effects of statins in other subgroups

Additional file 4 shows the results of traditional meta-analysis. For patients with T2DM, SRP + statins showed additional benefits in IBD fill (WMD: 1.39 mm; 95% CI: 1.25–1.53; I^2^ = 0.0%), PD reduction (WMD: 2.37 mm; 95% CI: 1.97–2.78; I^2^ = 0.0%%), and CAL gain (WMD: 2.69 mm; 95% CI: 2.26, 3.12; I^2^ = 0.0%). For smokers, significantly greater benefits were observed with SRP + statins treatment for IBD (WMD: 1.35 mm; 95% CI: 1.24–1.46; I^2^ = 0.0%), PD (WMD: 2.62 mm; 95% CI: 1.97–3.28; I^2^ = 67.2%), and CAL (WMD: 2.18 mm; 95% CI: 1.72–2.64; I^2^ = 0.0%).

### Evaluation of consistency and fit of the models

The results of pairwise and network meta-analysis are presented in Additional file 5 and Fig. 3. The effect size and relevant CI or CrI were found to be similar between pairwise and network meta-analyses. The result of node-splitting analysis showed no inconsistency (Additional file 6) and the data was well-fitted to the model with Dbar approximation of the data points in PD reduction, CAL gain and IBD fill (Additional file 5).

### Sensitivity analysis

After excluding three studies with high risk of bias [28, 29, 41], the results were not significantly altered (Additional file 6).

## Discussion

Statins, possess anti-inflammatory, anti-microbial, osteo-simulative, and antioxidant properties which may partly account for their beneficial effects in treating CP. Statins were shown to suppress inflammatory factors associated with periodontitis such as IL-6, TNF-α [43], IL-1β [44], as well as periodontal pathogens *Pg* and *Aa* [45]. Statins could also inhibit the secretion of matrix metalloproteinases (MMPs) [46], which are involved in the destruction of periodontal tissue. Moreover, statins increase bone regeneration by inducing the expression of BMP-2, VEGF, and OPG [47, 48]. Thus, it is unsurprising that local use of statins provides additional benefits for periodontal parameters of CP with or without systemic disease.

Traditional meta-analyses fail to measure the relative effect as they only synthesize studies with the same pair of comparators; network meta-analyses have been proposed to overcome this drawback. In our study, we performed a Bayesian network meta-analysis to compare the relative effect of different statins and found their efficacy to be similar, consistent with a study by Muniz et al. [20] who used meta-regression. Contrastingly, a study by Bertl et al. [4] found that RSV was more efficacious than SMV for all parameters tested and ATV in all parameters except for residual IBD. However, Bertl et al. [4] included patients with different characteristics and different periodontal therapy which may partially account for this inconsistency with our results. More direct evidence is needed in further test and compare the efficacy of different statins.

In addition, another advantage of network meta-analysis is that Bayesian chain assists in ranking the treatment efficacy by measuring the corresponding probability [49], so that it could provide more evidence to guide clinicians. Though we found no difference between diverse statins, ranking can pave the way for understanding the differences in opinions on the use of either statin in periodontal disease. Our results indicate that SMV is ranked the best in PD reduction and CAL gain. SMV is considered to be the best statin against periodontal pathogens such as *Pg* and *Aa*. Moreover, SMV was observed to decrease the expression of MMP-1, MMP-3, MMP-8, MMP-9 and MMP-13 [50–52]. RSV may be the best optimal performer in terms of IBD fill. Additionally, RSV has a greater anti-inflammatory action due to more effective suppression of C-reactive protein levels. Moreover, RSV is more effective in reducing low-density lipoprotein cholesterol which had benefits in induced periodontitis in hypertensive rats via inflammatory gene profile modulation [53].

We also assessed the efficacy of adjunctive statins in CP with T2DM comorbidity or smoking history as these are both risk factors for CP. High levels of blood-glucose increase advanced glycation end-products (AGE) and receptor of AGE (RAGE) leading to an exaggerated inflammatory response and periodontal tissue destruction by oxidative mechanisms [54, 55]. Smoking can similarly up-regulate the expression of RAGE [56, 57]. Statins possess strong antioxidant properties which may improve treatment outcomes for CP patients with T2DM or those who smoke. Existing RCTs indicate that locally applied ATV or SMV adjunctive to SRP was more effective than SRP alone in CP patients with T2DM or in smokers [32, 33, 35, 38]. The results of our traditional meta-analyses also support these findings and is consistent with another meta-analysis conducted by Ambrósio et al. [19]. However, the sample size in these trials was too small to draw a strong conclusion and more high-quality RCTs are needed to further to validate our results.

We observed a high degree of heterogeneity in CP patients without other systemic diseases. This may be attributable to variables such as different gel doses of statins used for treatment (0.1 ml or 10 ul) in the included trials. In addition, the measurement of IBD from the conventional radiographs was not calibrated which may have caused geometric errors in assessing IBD fill.

### Limitations

This network meta-analysis has several limitations that should be noted. Firstly, the length of follow-up of the included trials were relatively short. Secondly, the sample sizes (28–34) for each group were relatively small. Finally, the heterogeneity was high despite decreasing the discrepancy among the characteristics of patients. Multi-centered RCTs with larger sample size and with an extended follow-up duration up to 12 or 24 months are needed to confirm the beneficial effects of statins in combination with nonsurgical periodontal treatment for CP.

## Conclusions

Taken together, this meta-analysis shows that SRP + ATV/RSV/SMV confers additional benefits in treating CP by SRP. However, clinicians must be cautious in applying these conclusions as further studies are required for validation of these results.

## Additional files

Additional file 1: Search strategy used in PubMed/MEDLINE. (DOCX 14 kb)

Additional file 2: Outcomes of studies included in the network meta-analysis. (DOCX 22 kb)

Additional file 3: Risk of bias summary: review authors’ judgements about each risk of bias item for each included study. (DOCX 35 kb)

Additional file 4: Forest plot on the effect size of subgroups. (DOCX 688 kb)

Additional file 5: Evaluation of consistency and fit of the models. (DOCX 3596 kb)

Additional file 6: Sensitivity analysis of outcomes by excluding trials with a high risk of bias. (DOCX 148 kb)

## Data Availability

All data generated or analyzed during this study are included in this published article.

## Abbreviations

Aa: Aggregatibacter actinomycetemcomitans
AD: Absolute difference
AGE: Advanced glycation end-products
ATV: Atorvastatin
CAL: Clinical attachment level
CIs: Confidence intervals
CP: Chronic periodontitis
Dbar: Posterior mean residual deviance
IBD: Intrabony defect depth
MD: Mean difference
PD: Pocket depth
Pg: Porphyromonas gingivalis
RAGE: Receptor of AGE
RCT: Randomized controlled clinical trial
RSV: Rosuvastatin
SD: Standard deviations
SMV: Simvastatin
SRP: Scaling and root planning
T2DM: Type 2 diabetes mellitus
WMD: Weighted mean difference
