# Supplementary material for: Efficacy of locally-delivered statins adjunct to non-surgical periodontal therapy for chronic periodontitis: a Bayesian network analysis
Source: BMC Oral Health. 2019 Jun 13;19:105. doi: 10.1186/s12903-019-0789-2 (PMC6567452; doi:10.1186/s12903-019-0789-2)
Supplement: Supplementary file 1 — Search strategy used in PubMed/MEDLINE. (DOCX 14 kb) [file 12903_2019_789_MOESM1_ESM.docx]

**Additional file 1: Table S1** Search strategy used in PubMed/MEDLINE

|  | Search terms |
| --- | --- |
| No. 4 | No 1 and No 2 and No 3 |
| No. 3 | (randomized controlled trial[Publication Type] OR randomized [Title/Abstract] OR  placebo[Title/Abstract]) |
| No. 2 | (((((((("Hydroxymethylglutaryl-CoA Reductase Inhibitors"[Mesh]) OR ((statin*) OR HMG-CoA Reductase Inhibitors))) OR (((atorvastatin) OR fluvastatin) OR rosuvastatin OR simvastatin))) OR ((3-hydroxy-3-methylglutaryl co-enzyme A reductase inhibitors) OR hydroxyl methylglutaryl-coenzyme A reductase inhibitors))) |
| No. 1 | ((((((((((Periodontitis[MeSH Terms]) OR Chronic Periodontitis[MeSH Terms]) OR periodontal diseases) OR periodontal pocket) OR attachment loss) OR alveolar bone loss) OR furcation) OR intrabony) OR infrabony) OR bone regeneration)) |
