# Supplementary material for: Efficacy of locally-delivered statins adjunct to non-surgical periodontal therapy for chronic periodontitis: a Bayesian network analysis
Source: BMC Oral Health. 2019 Jun 13;19:105. doi: 10.1186/s12903-019-0789-2 (PMC6567452; doi:10.1186/s12903-019-0789-2)
Supplement: Supplementary file 2 — Outcomes of studies included in the network meta-analysis. (DOCX 22 kb) [file 12903_2019_789_MOESM2_ESM.docx]

| *Additional file 3: Table S3*: Outcomes of included studies | | | | | | | | |
| --- | --- | --- | --- | --- | --- | --- | --- | --- |
| Study  (year) | Periodontal probe | B-PD  (T vs. C) | B-CAL  (T vs. C) | B-IBD  (T vs. C) | Follow-up  (m) | C-PD  (T vs. C) | C-CAL  (T vs. C) | C-IBD  (T vs. C) |
| Pradeep  (2010) | UNC-15 periodontal probe, Hu-Friedy, Chicago, IL | 7.43±1.59 vs. 6.87±1.61 | 6.23±1.45 vs. 6.20±1.65 | 4.35±1.14 vs. 4.38±1.19 | 6 | 1m: 3.03±0.99 vs. 1.73±1.11  2m: 3.80±1.09 vs. 1.66±1.02  4m: 4.23±1.33 vs. 1.66±1.24  6m: 4.26±1.59 vs. 1.20±1.24 | 1m: 2.73±1.70 vs. 1.46±1.73  2m: 3.43±1.71 vs. 1.76±1.90  4m: 3.96±1.75 vs. 1.73±1.98  6m: 4.36±1.92 vs. 1.63±1.99 | 6m: 1.41±0.74 vs. 0.09±0.58 |
| Pradeep  (2012) | UNC-15 periodontal probe, Hu-Friedy, Chicago, IL | 7.33±1.49 vs. 6.80±1.32 | CAL:7.92±1.50 vs. 7.43±1.53  RHAL: 8.43±1.28 vs. 7.86±1.46 | 4.57±1.14 vs. 4.33±0.99 | 6 | 3m: 3.06±1.09 vs. 1.66±1.19  6m: 4.05±1.31 vs. 1.30±1.01 | CAL: 3m: 3.70±1.21 vs. 2.36±1.25  6m: 4.63±1.01 vs. 2.46±1.49  RHAL: 3m: 3.50±1.28 vs. 2.30±1.75  6m: 4.33±1.42 vs. 2.43±1.66 | 6m: 1.15±0.61 vs 1.15±0.61 |
| Rath  (2012) | Not report | 6.3±1.7 vs. 6.6±1.5 | 7.5±1.6 vs. 7.4±1.0 | 7.2±1.2 vs. 7.3±1.1 | 6 | 2m: 1.9±1.7 vs. 1.1±0.5  3m: 3.1±1.5 vs. 1.8±0.7  6m: 4.0±1.6 vs. 2.1±0.8 | 2m: 2.2±1.5 vs. 1.4±1.1  3m: 3.6±1.6 vs. 2±1.4  6m: 4.6±1.5 vs. 2.3±1.1 | 6m: 0.57±1.0 vs. 0.08±0.1 |
| Pradeep  (2013a) | UNC-15 periodontal probe, Hu-Friedy, Chicago, IL | 7.77±1.30 vs. 7.73±1.14 | 7.03±1.43 vs. 7.40±1.22 | 4.79±0.54 vs. 4.75±0.51 | 9 | 3m: 2.20±0.54 vs. 1.36±0.54  6m: 3.40±0.56 vs. 1.56±0.53  9m: 3.70±0.59 vs. 1.46±0.55 | 3m: 2.70±0.54 vs. 1.86±0.55  6m: 4.20±0.60 vs. 2.36±0.51  9m: 4.46±0.60 vs. 2.26±0.51 | 6m: 1.60±0.24 vs. 0.13±0.25  9m: 1.70±0.24 vs. 0.08±0.26 |
| Pradeep  (2013b) | UNC-15 periodontal probe, Hu-Friedy, Chicago, IL | 8.31±1.04 vs. 7.86±1.09 | 6.41±0.87 vs. 6.31±0.81 | 4.89±0.51 vs. 4.92±0.56 | 9 | 3m: 2.79±1.18 vs. 1.48±0.57  6m: 3.79±1.15 vs. 1.69±0.76  9m: 4.03±1.27 vs. 1.55±0.95 | 3m: 2.48±0.74 vs. 1.00±0.27  6m: 3.83±0.97 vs. 1.38±0.56  9m: 3.97±1.18 vs. 1.14±0.83 | 6m: 1.38±0.73 vs. 0.19±0.37  9m: 1.62±0.71 vs. 0.23±0.49 |

| Study  (year) | Periodontal probe | B-PD  (T vs. C) | B-CAL  (T vs. C) | B-IBD  (T vs. C) | Follow-up  (m) | C-PD  (T vs. C) | C-CAL  (T vs. C) | C-IBD  (T vs. C) |
| --- | --- | --- | --- | --- | --- | --- | --- | --- |
| Rao  (2013) | UNC-15 periodontal probe, Hu-Friedy, Chicago, IL | 7.87±0.90 vs. 7.93±1.12 | 6.20±1.16 vs. 6.13± 0.94 | 4.75±0.85 vs. 4.86± 0.46 | 9 | 3m: 2.30±1.06 vs. 1.23±1.01  6m: 3.37±1.27 vs. 1.90±1.32  9m: 3.83±1.26 vs. 1.57±1.22 | 3m: 2.13±0.94 vs. 1.00±0.79  6m: 3.20±1.32 vs. 1.67±1.18  9m: 3.63±1.10 vs. 1.47±1.41 | 6m: 1.17±0.45 vs. 0.13±0.26  9m: 1.51±0.47 vs. 0.21±0.27 |
| Pradeep  (2015) | UNC-15 periodontal probe, Hu-Friedy, Chicago, IL | 7.08±0.74 vs. 7.05± 0.65 | 6.27±0.63 vs. 6.24±0.49 | 4.59±0.62 vs. 4.59±0.74 | 6 | 1m: 3.14±0.28 vs. 2.60±0.08  3m: 3.31±0.28 vs. 2.27±0.1  4m: 3.68±0.3 vs. 1.78±0.07  6m: 4.04±0.34 vs. 1.31±0.24 | 1m: 2.31±0.04 vs. 1.98±0.19  3m: 2.92±0.07 vs. 1.68±0.14  4m: 3.61±0.23 vs. 1.68±0.16  6m: 4.20±0.17 vs. 1.40±0.15 | 6m: 2.23±0.32 vs. 0.46±0.02 |
| Kumari  (2016) | UNC-15 periodontal probe, Hu-Friedy, Chicago, IL | 8.17±1.12 vs. 8.03±1.19 | 7.10±1.16 vs. 6.93±1.08 | 4.84±0.47 vs. 4.80±0.47 | 9 | 3m: 2.43±0.85 vs. 1.33±0.80  6m: 3.56±1.10 vs. 1.66±0.95  9m: 3.80±1.18 vs. 1.53±1.04 | 3m: 2.46±1.13 vs. 1.2±1.03  6m: 3.63±1.40 vs. 1.46±1.50  9m: 3.76±1.43 vs. 1.36±1.54 | 6m: 1.42±0.44 vs. 0.17±0.12  9m: 1.51±0.44 vs. 0.12±0.10 |
| Pradeep  (2016) | UNC-15 periodontal probe, Hu-Friedy, Chicago, IL | I: 7.37 ± 0.62  II: 7.29 ± 0.66  III: 7.29 ± 0.60 | I: 5.37 ± 0.62  II: 5.29 ± 0.66  III: 5.29 ± 0.60 | I: 5.98 ± 0.35  II: 5.96 ± 0.19  III: 5.92 ± 0.26 | 9 | 6m: I: 3.03 ± 0.43  II: 2.33 ± 0.48  III: 1.47 ± 0.50  6-9m: I: 1.55 ± 0.64  II: 1.03 ± 0.51  III: 0.44 ± 0.50 | 6m: I: 2.88 ± 0.42  II: 2.33 ± 0.48  III: 1.37 ± 0.49  6-9m: I: 1.40 ± 0.50  II: 1.00 ± 0.48  III: 0.48 ± 0.50 | 6m: I: 2.83 ± 0.53  II: 2.29 ± 1.06  III: 0.07 ± 0.26  6-9m: I: 1.48 ± 0.57  II: 1.03 ± 0.19  III: 0.03 ± 0.19 |

| Study  (year) | Periodontal probe | B-PD  (T vs. C) | B-CAL  (T vs. C) | B-IBD  (T vs. C) | Follow-up  (m) | C-PD  (T vs. C) | C-CAL  (T vs. C) | C-IBD  (T vs. C) |
| --- | --- | --- | --- | --- | --- | --- | --- | --- |
| Garg  (2017) | UNC-15 periodontal probe, Hu-Friedy, Chicago, IL | 7.36±1.12 vs. 7.23±1.25 vs. 7.63±1.06 | CAL: 8.56±1.22 vs. 8.83±1.11 vs. 8.86±1.07  RHAL: 8.26±1.04 vs. 8.43±1.13 vs. 8.36±0.99 | 4.21±0.21 vs. 4.07±0.31 vs. 4.10±0.38 | 9 | 6m: 3.3±0.46 vs. 2.43±0.62 vs. 1.63±0.49  9m: 4.33±0.54 vs. 3.10±0.75  vs. 1.83±0.87 | CAL: 6m: 3.63±0.55 vs. 3.00±0.58 vs. 1.80±0.40  9m: 4.50±0.73 vs. 3.86±0.43 vs. 2.06±0.52  RHAL: 6m: 3.23±0.55 vs. 2.70±0.46 vs. 1.86±0.62  9m: 3.93±0.73 vs. 3.33±0.54 vs. 2.10±0.48 | 6m: 1.31±0.42 vs. 1.06±0.43 vs. 0.196±0.15  9m: 1.77±0.39 vs. 1.42±0.44 vs. 0.213±0.16 |
| Kumari  (2017) | UNC-15 periodontal probe, Hu-Friedy, Chicago, IL | 6.97±1.44 vs. 7.03±1.38 | 6.90±1.40 vs. 6.94±1.41 | 4.73±0.54 vs. 4.71±0.52 | 9 | 3m: 1.54±1.17 vs. 0.88±0.60  6m: 2.66±1.43 vs. 1.00±0.93  9m: 3.84±0.83 vs. 0.91±1.07 | 3m: 2.27±1.07 vs. 1.48±0.97  6m: 3.61±1.41 vs. 1.91±1.24  9m: 4.06±1.64 vs. 1.85±1.28 | 6m: 1.44±0.41 vs. 0.14±0.09  9m: 1.53±0.40 vs. 0.15±0.13 |
| Pradeep  (2017) | UNC-15 periodontal probe, Hu-Friedy, Chicago, IL | 6.56±1.38 vs. 6.76±1.22 | 6.13±0.89 vs. 6.13±1.13 | 5.46±0.81 vs. 5.17±0.35 | 9 | 3m: 1.06±0.63 vs. 0.33±0.47  6m: 2.46±0.97 vs. 1.06±0.90  9m: 3.53±1.27 vs. 1.50±1.07 | 3m: 1.66±0.60 vs. 0.16±0.37  6m: 3.7±0.91 vs. 1.16±0.94  9m: 4.26±1.08 vs. 1.50±1.07 | 6m: 1.90±0.44 vs. 0.09±0.17  9m: 1.98±0.72 vs. 0.12±0.17 |
| Martande  (2017) | UNC-15 periodontal probe, Hu-Friedy, Chicago, IL | 7.53 ± 1.35 vs. 7.70 ± 1.29 vs. 7.63 ± 1.18 | 6.70 ± 1.17 vs. 6.83 ± 1.14 vs. 6.83 ± 1.01 | 4.86 ± 0.56 vs. 4.75 ± 0.50 vs. 4.72 ± 0.47 | 9 | 3m: 2.2±1.19 vs. 1.97±1.14 vs. 1.17±1.64  6m: 3.08±1.17 vs. 2.97±1.14 vs. 1.55±1.63  9m: 3.37 ± 1.32 vs. 3.10 ± 1.55 vs. 1.25 ± 1.24 | 3m: 2.24±1.09 vs. 2.1±1.08 vs. 1.2±1.00  6m: 3.34±1.07 vs. 3.07±1.23 vs. 1.43±1.04  9m: 3.46 ± 1.47 vs. 3.13 ± 1.56 vs. 1.30 ± 1.51 | 6m: 1.62±0.52 vs. 1.45±0.47 vs. 0.16±0.45  9m: 1.69 ± 0.34 vs. 1.53 ± 0.41 vs. 0.12 ± 0.10 |

| Study  (year) | Periodontal probe | B-PD  (T vs. C) | B-CAL  (T vs. C) | B-IBD  (T vs. C) | Follow-up  (m) | C-PD  (T vs. C) | C-CAL  (T vs. C) | C-IBD  (T vs. C) |
| --- | --- | --- | --- | --- | --- | --- | --- | --- |
| DILEEP P  (2018) | UNC 15 color-coded periodontal probe, Hu-Friedy, Chicago, IL | 7.30 ± 1.17 vs. 6.80 ± 1.21 | 6.23 ± 1.01 vs. 6.06 ± 1.17 | 5.26 ± 0.87 vs. 5.15 ± 0.35 | 12 | 6m: 2.661 ± .844 vs. 1.033 ± 0.76  12m: 3.733 ± 1.284 vs. 1.533 ± 1.074 | 6m: 2.766 ± 1.135 vs. 1.233 ± 0.897  12m: 3.900 ± 1.155 vs. 1.4667 ± 1.074 | 6m: 1.523 ± 0.780 vs. 0.0767 ± 0.199  12m: 2.173 ± 0.784 vs. 0.1067 ± 0.199 |

B-PD, baseline value of probing depth; B-CAL, baseline value of clinical attachment loss; B-IBD, baseline value of intrabony defect; C-PD, change value of probing depth; C-CAL, change value of clinical attachment loss; C-IBD, change value of intrabony defect; SMV, simvastatin; ATV, atorvastatin; RSV, rosuvastatin
