# Supplementary material for: Efficacy of locally-delivered statins adjunct to non-surgical periodontal therapy for chronic periodontitis: a Bayesian network analysis
Source: BMC Oral Health. 2019 Jun 13;19:105. doi: 10.1186/s12903-019-0789-2 (PMC6567452; doi:10.1186/s12903-019-0789-2)
Supplement: Supplementary file 3 — Risk of bias summary: review authors’ judgements about each risk of bias item for each included study. (DOCX 35 kb) [file 12903_2019_789_MOESM3_ESM.docx]

*Additional file 4: Table S4*: *Risk of bias of studies on non-surgical and surgical periodontal treatment according to the Cochrane Collaboration‘s Tool (Higgins & Green 2011).*

| First author,  year | Random sequence generation | Allocation concealment | Blinding of participants and personnel | Blinding of outcome assessment | Incomplete outcome data | Selective outcome reporting | Other bias | Overall risk of bias |
| --- | --- | --- | --- | --- | --- | --- | --- | --- |
| Pradeep (2010)^1^ | + | (random sequence generation and treatment was the same person) | + | + | + | + | + | _ |
| Pradeep (2012)^2^ | + | -  (random sequence generation and radiographic Evaluation was the same person) | + | + | + | + | + | - |
| Rath (2012)^3^ | + | ? (not report) | + | ? (assessment of periodontal parameters was not reported) | + | + | + | ? |
| Pradeep (2013a)^4^ | + | + | + | + | + | + | + | + |
| Pradeep (2013b)^5^ | + | + | + | + | + | + | + | + |
| Rao (2013)^6^ | + | + | + | + | + | + | + | + |
| Pradeep (2015)^7^ | + | + | + | + | + | + | + | + |
| Kumari (2016)^8^ | + | + | + | + | + | + | + | + |
| Pradeep (2016a)^9^ | + | ? (not report) | + | + | + | + | + | ? |
| Grag (2017)^10^ | + | + | + | + | + | + | + | + |
| Kumari (2017)^11^ | + | + | + | + | + | + | + | + |
| Pradeep (2017)^12^ | + | ? (not report) | + | + | + | + | + | ? |
| Martande (2017)^13^ | + | ? (not report) | + | ? (assessment of periodontal parameters was not reported) | + | + | + | ? |
| DILEEP P (2018)^14^ | + | (random sequence generation and treatment was the same person) | + | + | + | + | + | - |

“+”, low risk; “-“, high risk; “?”, unclear risk.

**References**

**1.** Pradeep AR, Thorat MS. Clinical effect of subgingivally delivered simvastatin in the treatment of patients with chronic periodontitis: a randomized clinical trial. *Journal of periodontology.* Vol 812010:214-222.

**2.** Pradeep AR, Priyanka N, Kalra N, Naik SB, Singh SP, Martande S. Clinical efficacy of subgingivally delivered 1.2-mg simvastatin in the treatment of individuals with Class II furcation defects: a randomized controlled clinical trial. *Journal of periodontology.* Vol 832012:1472-1479.

**3.** Rath A, Mahenra J, Thomas L, Sandhu M, Namasi A, Ramakrishna T. A clinical, radiological and IL-6 evaluation of subgingivally delivered simvastatin in the treatment of chronic periodontitis. *International Journal of Drug Delivery.* 2012;4:70-81.

**4.** Pradeep AR, Kumari M, Rao NS, Martande SS, Naik SB. Clinical efficacy of subgingivally delivered 1.2% atorvastatin in chronic periodontitis: a randomized controlled clinical trial. *Journal of periodontology.* Vol 842013:871-879.

**5.** Pradeep AR, Rao NS, Bajaj P, Kumari M. Efficacy of subgingivally delivered simvastatin in the treatment of patients with type 2 diabetes and chronic periodontitis: a randomized double-masked controlled clinical trial. *Journal of periodontology.* Vol 842013:24-31.

**6.** Rao NS, Pradeep AR, Bajaj P, Kumari M, Naik SB. Simvastatin local drug delivery in smokers with chronic periodontitis: a randomized controlled clinical trial. *Australian dental journal.* Vol 582013:156-162.

**7.** Pradeep AR, Karvekar S, Nagpal K, Patnaik K, Guruprasad CN, Kumaraswamy KM. Efficacy of locally delivered 1.2% rosuvastatin gel to non-surgical treatment of patients with chronic periodontitis: a randomized, placebo-controlled clinical trial. *Journal of periodontology.* Vol 862015:738-745.

**8.** Kumari M, Martande SS, Pradeep AR, Naik SB. Efficacy of Subgingivally Delivered 1.2% Atorvastatin in the Treatment of Chronic Periodontitis in Patients With Type 2 Diabetes Mellitus: A Randomized Controlled Clinical Trial. *Journal of Periodontology.* 2016;87:1278-1285.

**9.** Pradeep AR, Garg V, Kanoriya D, Singhal S. 1.2% Rosuvastatin Versus 1.2% Atorvastatin Gel Local Drug Delivery and Redelivery in Treatment of Intrabony Defects in Chronic Periodontitis: A Randomized Placebo-Controlled Clinical Trial. *Journal of Periodontology.* 2016;87:756-762.

**10.** Garg S, Pradeep AR. 1.2% Rosuvastatin and 1.2% Atorvastatin Gel Local Drug Delivery and Redelivery in the Treatment of Class II Furcation Defects: A Randomized Controlled Clinical Trial. *Journal of Periodontology.* 2017;88:259-265.

**11.** Kumari M, Martande SS, Pradeep AR. Subgingivally delivered 1.2% atorvastatin in the treatment of chronic periodontitis among smokers: a randomized, controlled clinical trial. *Journal of investigative and clinical dentistry.* 2017;8.

**12.** Pradeep AR, Kanoriya D, Singhal S, Garg V, Manohar B, Chatterjee A. Comparative evaluation of subgingivally delivered 1% alendronate versus 1.2% atorvastatin gel in treatment of chronic periodontitis: a randomized placebo-controlled clinical trial. *Journal of Investigative and Clinical Dentistry.* 2017;8.

**13.** S Martande S, Kumari M, Pradeep AR, Pal Singh S, Kumar Suke D. Comparative evaluation of efficacy of subgingivally delivered 1.2% Atorvastatin and 1.2% Simvastatin in the treatment of intrabony defects in chronic periodontitis: a randomized controlled trial. *Journal of dental research, dental clinics, dental prospects.* 2017;11:18-25.

**14.** P D, S I, Kurian IG, Pradeep AR. Comparative evaluation of subgingivally delivered 1.2% rosuvastatinand 1% metformin gelin treatment of intrabony defects in chronic periodontitis: A randomized controlled clinical trial. *J Periodontol.* 2018.
