# Supplementary material for: Efficacy of locally-delivered statins adjunct to non-surgical periodontal therapy for chronic periodontitis: a Bayesian network analysis
Source: BMC Oral Health. 2019 Jun 13;19:105. doi: 10.1186/s12903-019-0789-2 (PMC6567452; doi:10.1186/s12903-019-0789-2)

**Additional file 5: Table S5** Forest plot on the effect size of subgroups.

Forest plot on the effect size of CP without systemic diseases


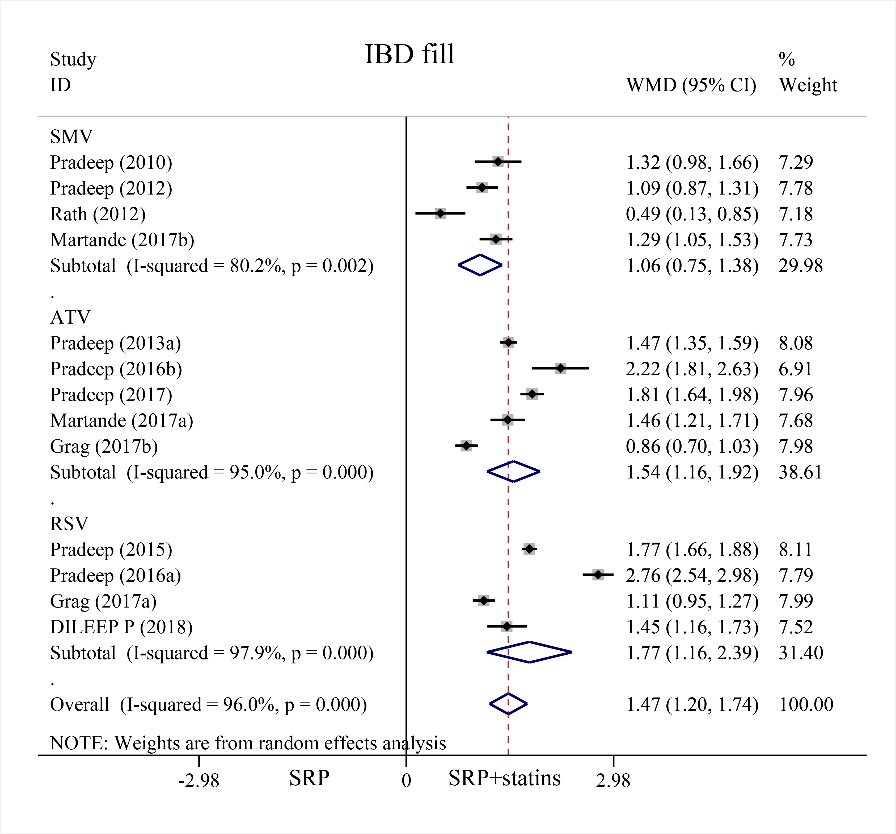


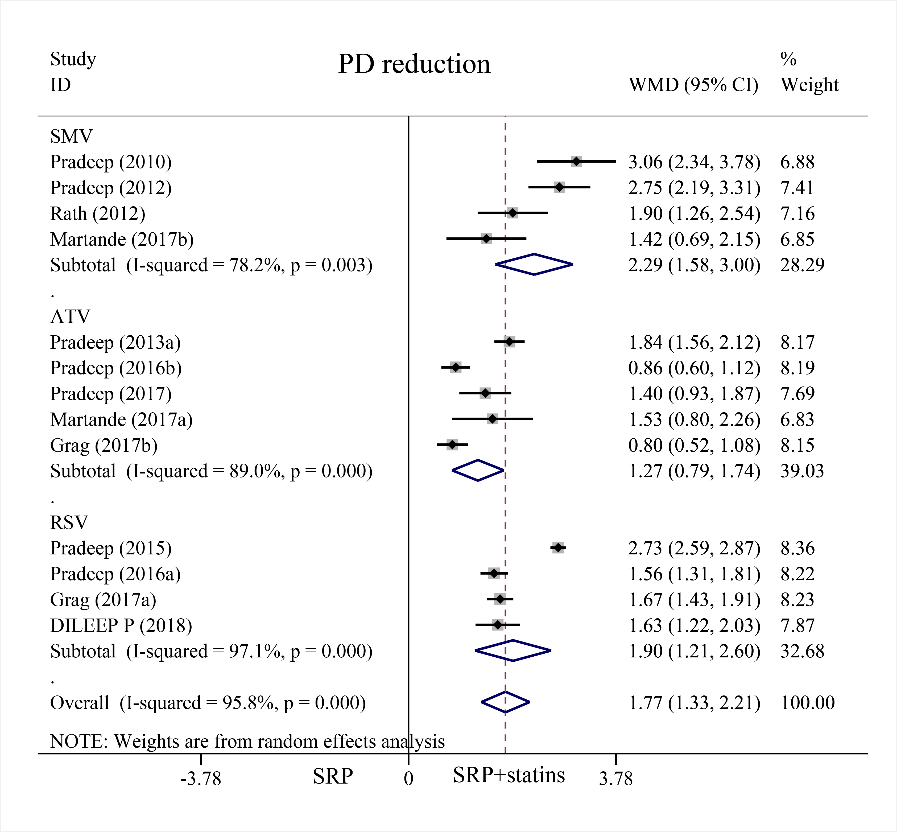


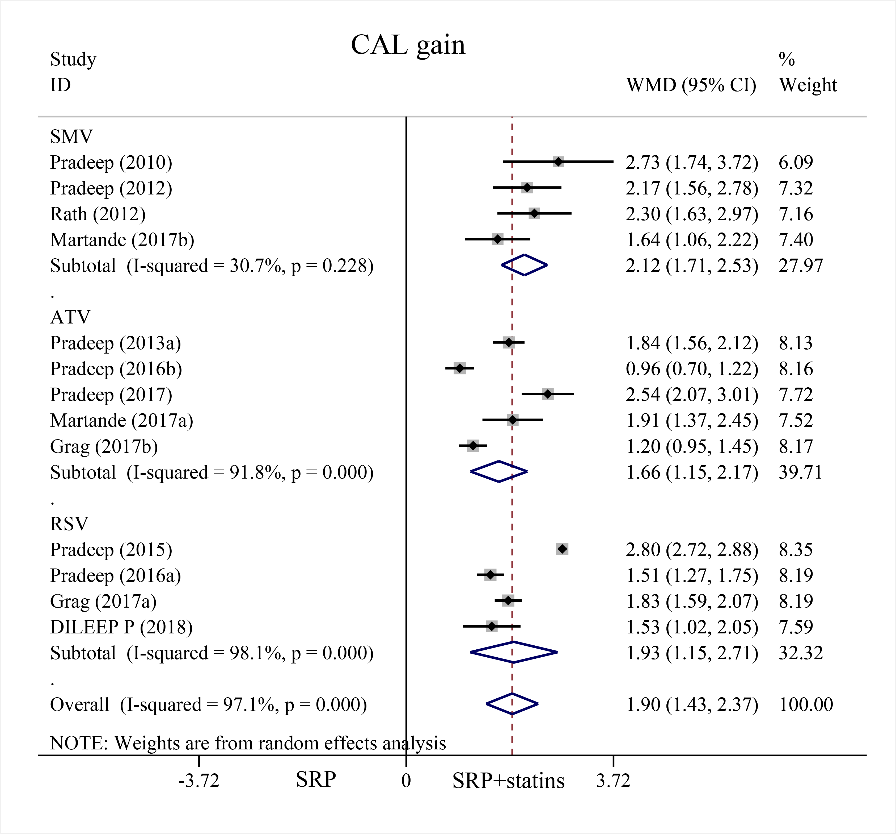


Forest plot on the effect size of CP with T2DM or smorking


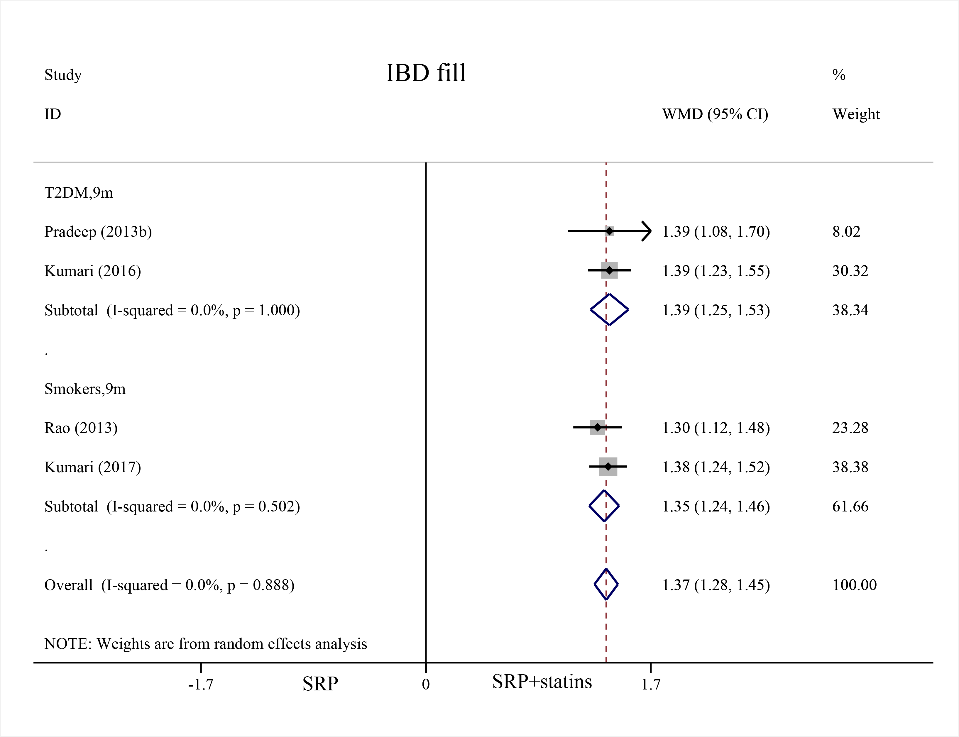


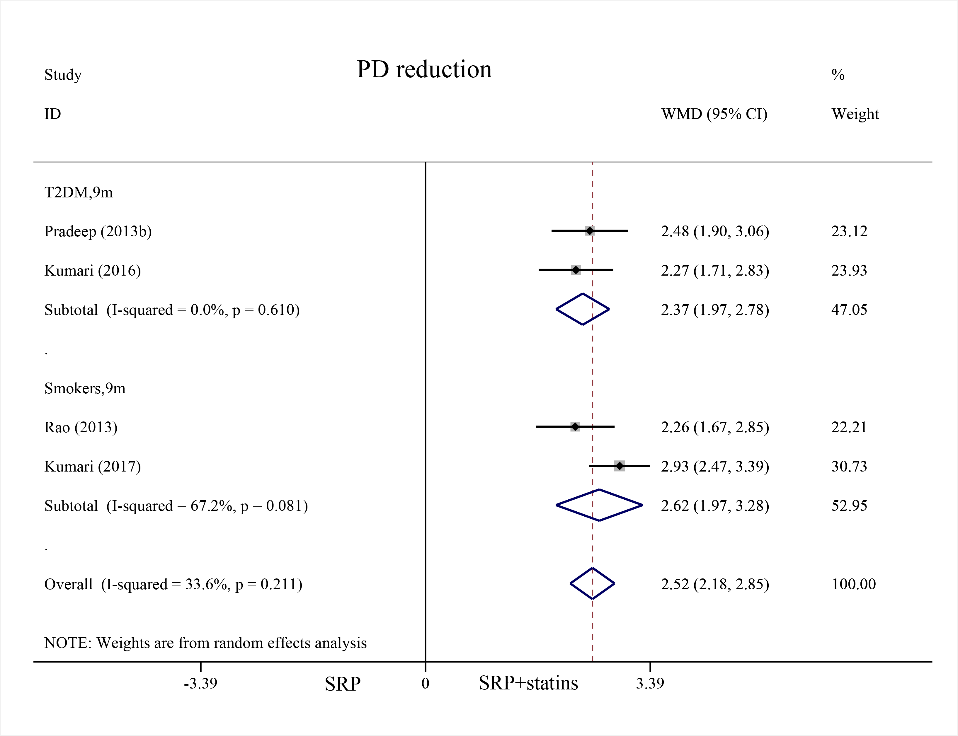


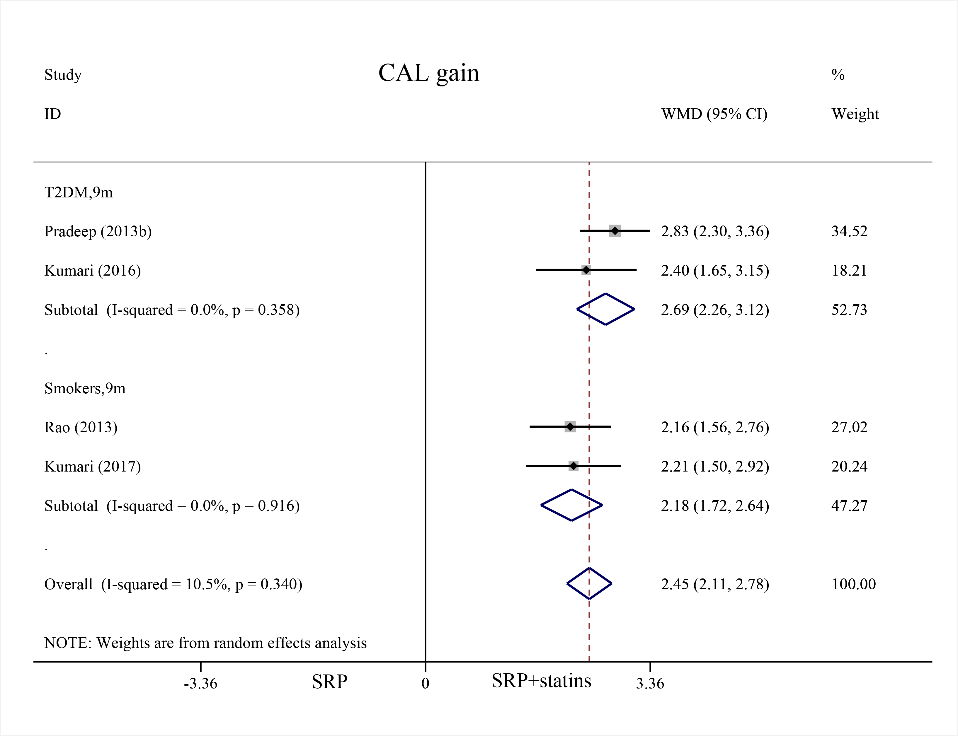

Supplement: Supplementary file 4 — Forest plot on the effect size of subgroups. (DOCX 688 kb) [file 12903_2019_789_MOESM4_ESM.docx]
