# Supplementary material for: Efficacy of locally-delivered statins adjunct to non-surgical periodontal therapy for chronic periodontitis: a Bayesian network analysis
Source: BMC Oral Health. 2019 Jun 13;19:105. doi: 10.1186/s12903-019-0789-2 (PMC6567452; doi:10.1186/s12903-019-0789-2)
Supplement: Supplementary file 5 — Evaluation of consistency and fit of the models. (DOCX 3596 kb) [file 12903_2019_789_MOESM5_ESM.docx]

**Additional file 6**. Evaluation of consistency and fit of the models.

(a) The results of node analysis

IBD fill


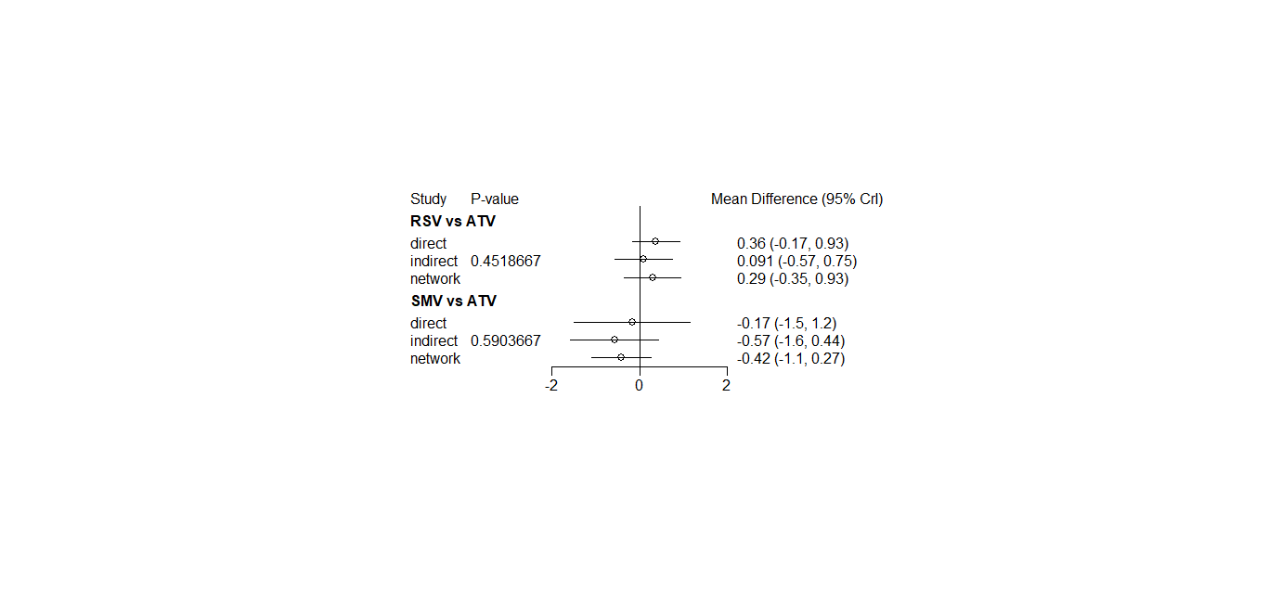


PD reduction


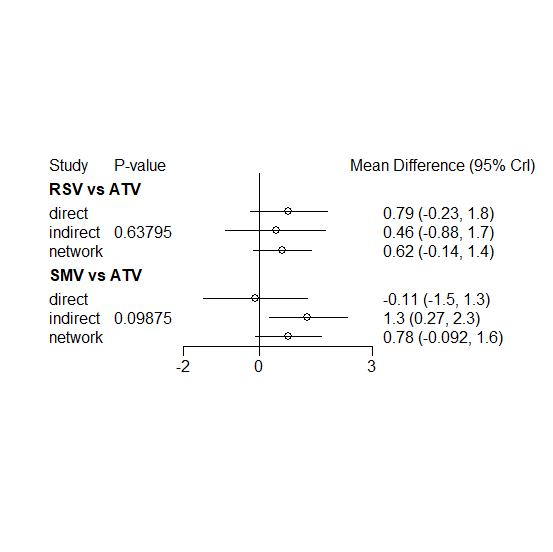


CAL gain


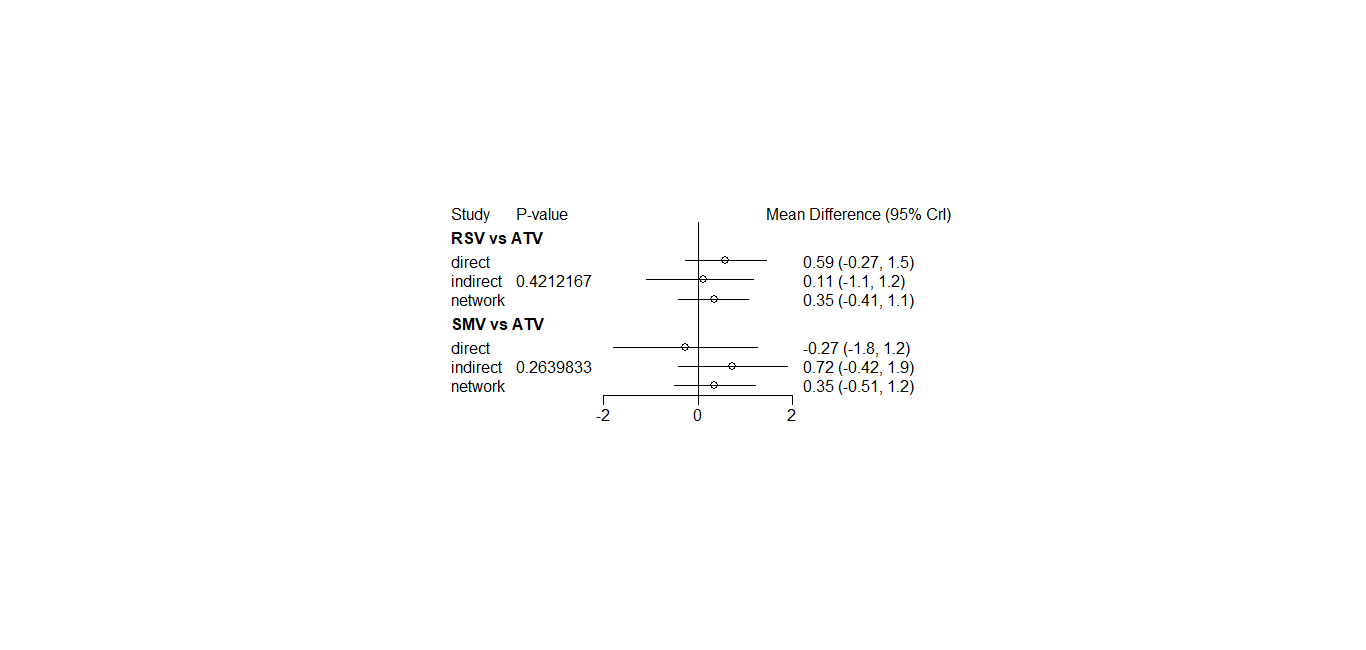


(b) Evaluation of model ft in the included studies

| Outcome | Dbar | Number of data points |
| --- | --- | --- |
| PD reduction | 23.17881 | 23 |
| CAL gain | 22.78038 | 23 |
| IBD fill | 22.96449 | 23 |

PD, probing depth; CAL, clinical attachment loss; IBD, intrabony defect; SRP, scaling and root planing; SMV, simvastatin; ATV, atorvastatin; RSV, rosuvastatin.
