# Supplementary figures and images for: Efficacy of locally-delivered statins adjunct to non-surgical periodontal therapy for chronic periodontitis: a Bayesian network analysis
Source: BMC Oral Health. 2019 Jun 13;19:105. doi: 10.1186/s12903-019-0789-2 (PMC6567452; doi:10.1186/s12903-019-0789-2)

**Additional file 7
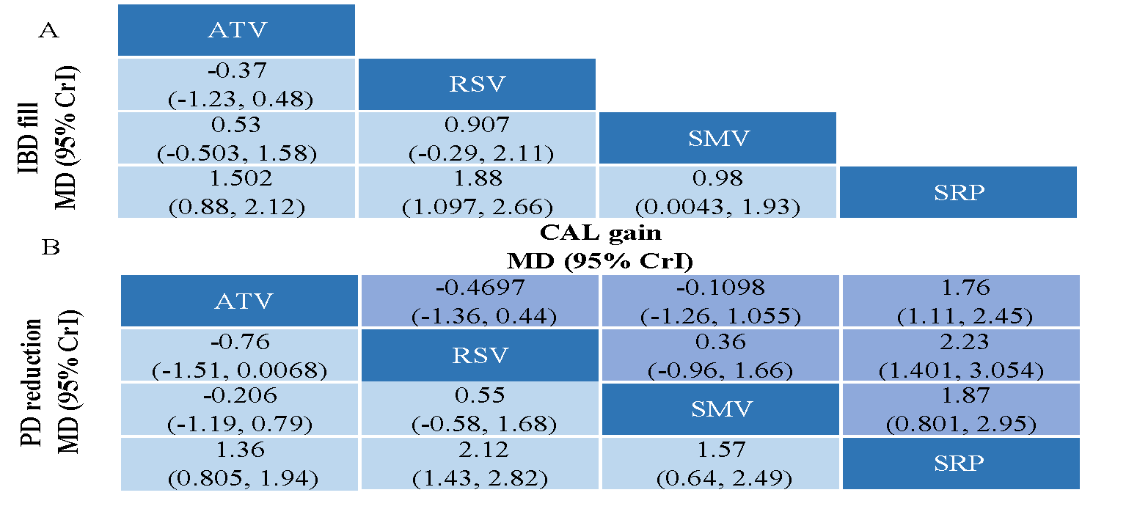
.** Sensitivity analysis of outcomes by excluding trials with a high risk of bias.

Supplement: Supplementary file 6 — Sensitivity analysis of outcomes by excluding trials with a high risk of bias. (DOCX 148 kb) [file 12903_2019_789_MOESM6_ESM.docx]
